# A quality-by-design optimized LC method for navigating degradation kinetics and quantification of favipiravir in the presence of degradation products and manufacturing impurities

**DOI:** 10.1186/s13065-025-01610-2

**Published:** 2025-08-15

**Authors:** Adel Ehab Ibrahim, Mohamed Farouk, Samy G. Alamir, Baher I. Salman, Tarek S. Belal, Sami El Deeb, Ahmed Al-Harrasi

**Affiliations:** 1 Natural and Medical Sciences Research Center, University of Nizwa, Birkat Al Mauz, 616 Nizwa City, Oman; 2 Pharmaceutical analytical chemistry department, Faculty of Pharmacy, Port-Said University, Port-Said, 42511 Egypt; 3 Nawah Scientific Inc, Mokatam, Cairo, Egypt; 4 Pharmaceutical Analytical Chemistry Department, Faculty of Pharmacy, Ain Shams University, Abassia, Cairo, 11566 Egypt; 5 Pharmaceutical Analytical Chemistry Department, Faculty of Pharmacy, Al-Azhar University, Assiut Branch, Assiut, 71524 Egypt; 6 Pharmaceutical Analytical Chemistry Department, Faculty of Pharmacy, University of Alexandria, Elmessalah, Alexandria, 21521 Egypt; 7 Institute of Medicinal and Pharmaceutical Chemistry, Technische Universitaet Braunschweig, 38106 Braunschweig, Germany

**Keywords:** Favipiravir, Degradation kinetics, Stability-indicating HPLC, Quality-by-design (QbD), Green chromatography, Impurity profiling

## Abstract

Emergent viruses require effective treatment regimens, and the severe acute respiratory syndrome coronavirus-2 variants are still evolving. Favipiravir has emerged as an effective antiviral medication, especially after its popularity against COVID-2019. Despite its growing popularity, favipiravir has not yet been included in any pharmacopeia, and new dosage forms are being developed to improve its bioavailability for various infections. Therefore, studying its stability and developing methods capable of detecting its impurities and degradation products are essential. An isocratic high-performance liquid chromatography with photodiode array detection method was also designed using the Quality-by-Design approach and validated to determine favipiravir in presence of its hydrolytic degradation products, besides two major manufacturing impurities (namely, 3,6-dichloro pyrazine-2-carbonitrile and 6-fluoro-3-hydroxypyrazine-2-carbonitrile). The separation was achieved using a mobile phase of 25.0 mM phosphate buffer (pH 3.04): acetonitrile (92: 8, v/v) at a 1.0 mL min^−1^ flow rate. A Hypersil C18-BDS column (5.0 μm, 250.0 × 4.6 mm) was employed at room temperature and detection at 323.0 nm. The method had a linear range (5.0–100.0 µg mL^−1^) with a limit of detection and quantification of 0.51 and 1.54 µg mL^−1^. The drug's stability after forced degradation and at different pH levels was explored. Key results proved that FAV is most stable at pH 5.0, with calculated activation energies for the acidic and alkaline degradation processes being 53492.276 and 61896.899 kJ/mole, respectively. Moreover, the analysis of expired favipiravir tablets revealed the presence of the alkaline degradate, thereby demonstrating the efficacy of the method. The proposed method was evaluated for its greenness and blueness to demonstrate its ecological safety and practicality, scoring 0.65 in the Analytical Greenness (AGREE) metric and 85.00 on the Blue Applicability Grade Index (BAGI). It is the first sustainable method developed using the Quality-by-Design to profile favipiravir together with its degradation products and impurities simultaneously.

**Supplementary Information:**

The online version contains supplementary material available at 10.1186/s13065-025-01610-2.

## Introduction

**Fig. 1:**
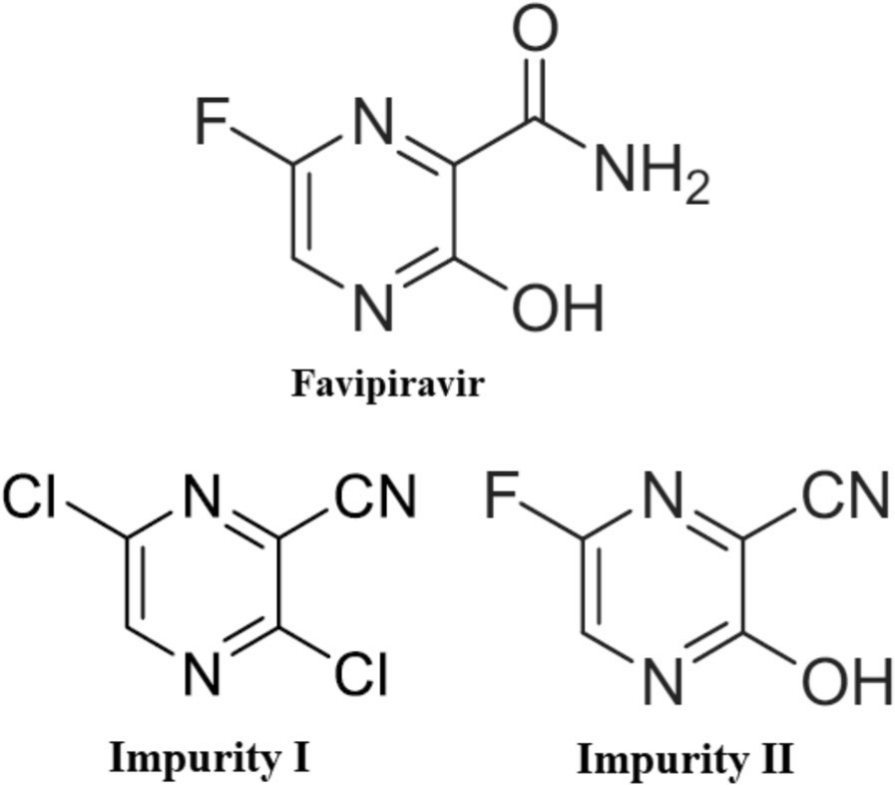
Chemical Structures of FAV, impurity (I) and impurity (II)

Coronavirus disease 2019 (COVID-19) is a contagious illness caused by the severe acute respiratory syndrome coronavirus 2 (SARS-CoV-2). To date, SARS-CoV-2 variants are still evolving [1]. Since there was no specific cure during the COVID-19 pandemic, healthcare providers had to rely on repurposing existing antiviral drugs [2]. One important medication that was extensively explored and incorporated in different regimens with other antiviral agents or with supportive treatments such as corticosteroids was Favipiravir (FAV) [3]. FAV, a broad-spectrum oral antiviral prodrug, was approved in Japan to treat drug-resistant influenza and marketed as the second-line treatment for novel or reemerging influenza outbreaks in China [4]. In addition to its success and efficacy in targeting several RNA viruses, such as influenza A and the yellow fever virus [2], Ebola [5], Junín [6], and Paramyxoviruses [7]. Besides, FAV improves virologic clearance, pneumonia symptoms, and reduces the need for supplemental oxygen and mechanical ventilation [8]. Moreover, recent studies have shown that FAV synergizes with Tamoxifen in treating Tamoxifen-resistant breast cancer [9]. Chemically named 6-fluoro-3-hydroxypyrazine-2-carboxamide, FAV (molecular weight 157.1; Fig. 1) is converted intracellularly to Favipiravir ribofuranosyl-5′-triphosphate (FAV-RTP). The FAV-RTP selectively inhibits viral RNA polymerase by acting as a competitive purine analog, preventing viral genome replication [10]. FAV is generally well-tolerated, but transient hyperuricemia and an increase in liver enzymes were commonly reported as adverse effects [11, 12].

The quality of drugs depends not only on their efficacy but also on their safety. As a result, the definition of quality in pharmaceuticals has shifted from ‘only purity’ to ‘impurity profiling and stability’ in finished products. Furthermore, international regulatory agencies have established comprehensive guidelines for this purpose, including the International Council for Harmonization (ICH): Q1A (R2), Q1C, Q3A (R2), and Q3B (R2) [13–15]. Additionally, various pharmacopeias, such as the United States Pharmacopeia and the British Pharmacopoeia, have updated their monograph elaborations to include impurity testing in accordance with guidelines set forth by international regulatory bodies [16–18]. Therefore, since favipiravir is still being included in new therapeutic regimens and there are no pharmacopeia monographs available, it necessitates the development of a robust analytical method for stability assessment and impurity analysis [19, 20]. Two common process-related impurities, 3,6-dichloro pyrazine-2-carbonitrile (Impurity I; molecular weight 173.9) and 6-fluoro-3-hydroxypyrazine-2-carbonitrile (Impurity II; molecular weight 139.1), originate from the FAV synthetic pathways. Impurity I can be the intermediate product in 6 out of 8 pathways using different starting materials [21]. It can also be used as the starting material in 4 pathways, where it undergoes fluorination with KF, resulting in a 3,6-difluoro intermediate. When the 3,6-difluoro intermediate is treated with sodium/potassium acetate, it produces Impurity II. Impurity II can then be converted into FAV through two of the four pathways [21]. Furthermore, Impurity I and II have been identified as intermediates in other pathways [19, 20]. According to batch analysis records from a manufacturer, impurities are determined using a 90 min gradient high-performance liquid chromatographic (HPLC) method [20]. The chemical structures of FAV and its studied impurities are shown in Fig. 1. To help achieve the safety goal, an analytical method must be designed to determine FAV, its impurities and degradation products. FAV's most stable conditions, such as pH, should also be identified.

Several methods have been published for the determination of FAV in different matrices. However, up to our knowledge, only a few methodologies were reported to identify FAV with its degradation and/or synthesis impurities. Some methods were established for determining the stability of FAV using HPLC–UV [19, 22–27], UV spectrophotometry [28], and LC–MS [29–32] techniques. Few methods were published for impurity profiling of FAV, including UV spectrophotometry [20, 33, 34], HPLC–UV [35] and HPLC–MS methods [30]. Remarkably, the FAV degradation kinetics were investigated in only one research paper [24], despite the fact that the degradation kinetics study of novel drugs is essential for ensuring their stability, safety, efficacy, and compliance with regulatory standards [36]. Most of these reported methods are stability-indicating assays that focus on forced stability (one or more of the acid, base, and oxidative stress conditions) or impurity detection, but not both simultaneously. Notably, Vemuri et al. [30] exerted remarkable efforts to profile FAV degradation products and certain FAV-related impurities using LC–MS; however, the chromatographic separation of degradation products was performed in separate runs. Still, it is worth noting that their method showed excellent sensitivity, achieving a limit of quantification as low as 0.09 μg mL^−1^.

One-factor-at-a-time (OFAT) is a sequential process of studying one factor while keeping other variables fixed, making it challenging to estimate interactions among the factors. Many of the reported methodologies relied on the OFAT approach during the development and optimization phases, without employing the design of experiments to examine the interactions and cubic effects of various parameters and to elucidate their influence on responses further. Hence, in recent years, Design of Experiments (DOE) approaches have been favored due to their efficiency in addressing the limitations of the OFAT approach and in achieving Quality by Design (QbD). [37, 38]. Moreover, by saving time and decreasing experimentation, DOE also reduces ecological and operator risk, aligning with the principles of green analytical chemistry [39]. Lastly, to our knowledge, no method has yet been reported to determine FAV in the presence of both its hydrolytic degradation products and process-related impurities in the same run. Additionally, most reported methods focus on Favipiravir quantification in pharmaceutical and biological matrices, overlooking ecological and practical aspects of the method.

To fill the gaps discussed above, this work aims to develop an optimized stability-indicating HPLC method using DOE to qualitatively and quantitatively determine FAV in the presence of its acidic and alkaline degradation products along with two very well-known manufacturing impurities (I and II). Another objective of the proposed research was to examine the kinetics of FAV degradation processes and determine the activation energies and the most stable pH for FAV, which -up to our knowledge- hasn’t been explored yet, to aid in developing new, effective, and stable dosage forms. The proposed method's environmental impact was assessed using the Analytical Greenness (AGREE) metric [40], and its feasibility was evaluated using the Blue Applicability Grade Index (BAGI) [41].

## Experimental

### Instruments

Chromatographic separations were performed using a Waters HPLC 2695 series system, connected to a Waters 2996 photodiode array detector (PDA) (Waters, USA). Empower-3 software was used for data collection and analysis, while Design Expert^®^ version 12 software (M/s Stat-Ease Inc., MN, USA) was employed for DOE. Hypersil C18-BDS column (5.0 μm, 250.0 × 4.6 mm) supplied by Thermo-Fisher (Massachusetts, USA) was used for separations and method validation. The pH of the mobile phase was adjusted using a bench-top pH-2005 m (SELECTA, Barcelona, Spain).

### Materials and reagents

The analytical standards of FAV (certified as 99.99% pure), as well as the pharmaceutical impurities 3,6-Dichloropyrazine-2-carbonitrile (Impurity I) and 6-fluoro-3-hydroxypyrazine-2-carbonitrile (Impurity II), were all provided by the Egyptian International Pharmaceutical Industries Company (EIPICo, Tenth of Ramadan City, Egypt). Acetonitrile (CAS #: 75-05-8) and Methanol (CAS #: 67-56-1), of HPLC grade, were purchased from Merck (Darmstadt, Germany). Sodium hydroxide (CAS #: 1310-73-2), potassium dihydrogen phosphate (CAS #: 7778-77-0), fuming hydrochloric acid (CAS #: 67-56-1) (37% w/w), boric acid (CAS #: 10043-35-3), and glacial acetic acid (CAS #: 64-19-7) were all analytical grade reagents obtained from Sigma-Aldrich (St. Louis, USA). Water was prepared in-house using double glass distillation and filtration through a 0.45 μm membrane filter.

Anviziram^®^, the commercial Favipiravir tablet dosage form, was manufactured by Rameda (6th of October City, Egypt). Each tablet contains 200 mg Favipiravir along with tablet excipients consisting of povidone K30, hydroxypropyl cellulose, crosspovidone, colloidal silicon dioxide (Aerosil 200), and sodium stearyl fumarate.

### Design of experiment (DOE)

The method development strategy using DOE involves defining critical method attributes (CMA), critical method parameters (CMP), and Quality Target Method Profile (QTMP). The CMPs in the proposed study included the flow rate, percentage and pH of the aqueous mobile phase. A response surface methodology, mixed-level Taguchi, was employed to optimize the impact of CMPs on CMAs: Resolution between FAV and Impurity-I (1) (R_1_), Resolution between Favipiravir and Impurity-II (2) (R_2_), analysis time, capacity factor (K′), FAV’s number of theoretical plates (N), and peak symmetry as elaborated in Table 1. The goal of QTMP was to attain resolutions over four, N of over 4000, a symmetry equal to one, K′ (> 1.5), and a maximum run time of 14 min; The stricter QTPs were implemented to enhance robustness and facilitate its transfer. After conducting the 18 runs depicted by the DOE, the model’s p-value and F-value, the lack of fit’s p-value, the regression coefficient (R^2^), adjusted R^2^, and adequate precision (S/N ratio) ratified the significance of each CMA model. The experimental design space is shown in Table 1.

**Table 1:** Design space showing critical method parameter (CMP), critical method attributes (CMA), and quality target method profile (QTMP)

CMP	Range	CMA	QTMP
1- pH	3.0–6.0	1-Resolution (1): between Favipiravir and Impurity (I)	Not Less than (NLT) 4
		2-Resolution (2): between Impurity (I) and impurity (II)	NLT 4
2- Water %	92.0–98.0 98.0%	3-Analysis time	8–14 min
		4-Theoretical plates for Favipiravir peak	NLT 4000
3- Flow rate	1.0–1.2	5-Capacity factor	NLT 1.5
		6- Symmetry of Favipiravir peak	0.99–1.0

### Chromatographic conditions

A 25 mM phosphate buffer was prepared in water, and the pH was adjusted to 3.04 using orthophosphoric acid. The mobile phase was prepared by mixing acetonitrile and phosphate buffer in an 8:92 (v/v) ratio. It was filtered using a Millipore 0.45-μm disposable filter (Milford, MA, USA) and degassed before use. The flow rate was set at 1.0 mL min^−1^, and all determinations were performed at a column temperature of 25.0 °C. The injection volume was 30.0 μL, and the detector wavelength was adjusted to 323.0 nm.

### Preparation of standard solutions

The amount of 10.0 mg of FAV pure standard was weighed and dissolved in 10.0 mL mobile phase to prepare a 1000.0 μg mL^−1^ stock solution. Six standard solutions with 5.0, 10.0, 25.0, 50.0, 70.0, and 100.0 μg mL^−1^ concentrations were prepared by diluting the stock solution using the mobile phase. Additionally, separate stock solutions for FAV’s impurities I and II were prepared in methanol at concentrations of 10.0 μg mL^−1^.

### Preparation of the degradation products

Solutions containing stress degradation products were prepared from FAV under acidic and alkaline stress conditions. First, 50.0 mg of FAV was precisely weighed and dissolved in 10.0 mL of 1.0 M hydrochloric acid. It was then heated in a water bath at 80.0 °C for two days to induce acidic degradation. Another 50.0 mg of FAV was dissolved in 10.0 mL of 2.0 M sodium hydroxide and heated in a water bath at 45.0 °C for two days for alkaline degradation. The solutions were neutralized and completed to volume using the mobile phase. The degradation was considered complete when either the Ct/C0 ratio of the FAV peak was ≤ 0.10 or when the ratio was approximately that value, and subsequent additional hours resulted in no observable FAV peak.

### Method validation

The chromatographic method’s optimal conditions were selected from the DOE experimentation and then were validated as per ICH Q2(R2) guidelines [13]. Linearity, range, accuracy, precision, specificity, Limit of Detection (LOD), and Limit of Quantification (LOQ) were calculated using the prepared standards.

The linearity standards were prepared from the FAV stock at concentrations 5.0, 10.0, 25.0, 50.0, 70.0, and 100.0 μg mL^−1^. Standards were injected in triplicate. The average peak area was plotted against the corresponding concentration to construct the calibration plot. The LOD and LOQ were calculated based on the response standard deviation (σ) and the slope (S) using the Eqs. 3.3 σ/S and 10 σ/S, respectively.

Three quality control (QC) standard concentrations were prepared for accuracy and precision validation. FAV was spiked into a placebo solution containing (0.1 gm mL^−1^) of the commonly co-formulated excipients: Povidone K30, hydroxypropyl cellulose, crosspovidone, colloidal silicon dioxide (Aerosil 200), and sodium stearyl fumarate. Three replicates of 25.0, 50.0, and 75.0 μg mL^−1^ concentrations were used for accuracy, and 10.0, 50.0, and 90.0 μg mL^−1^ for precision assessment through repeatability and inter-day precision (three consecutive days). The accuracy and precision were expressed as average recovery percentage (R%) ± relative standard deviation percentage (RSD%). Small deliberate changes to the pH (± 0.1) were implemented to evaluate the robustness utilizing the mid-level QC sample (50.0 μg mL^−1^).

The assessment of specificity was conducted by injecting a diluent blank, a placebo (excipient matrix) solution, the standard solution of FAV, the standard solution of impurities, and forced degradation samples. Additionally, the PDA detector was used to evaluate the purity of FAV peaks by examining the purity angle and purity threshold across all stressed samples.

To study the stability of the solutions, standard solutions of Favipiravir (50 µg/mL) and Favipiravir (50 µg/mL) with Impurity I & II (5 µg/mL) were stored in the HPLC autosampler rack in tightly sealed HPLC vials. These solutions were subsequently reinjected after 24 h, and new measurements were compared to the initial ones (0 h vs 24 h). Additionally, the stock solutions kept in the refrigerator were evaluated after 72 h and compared to freshly prepared solutions.

### Kinetic investigation study

A stress study was conducted to determine the degradation kinetics of FAV under acidic and alkaline conditions. An accurately weighed 12.5 mg of FAV was dissolved in 250.0 mL distilled water to make a concentration of 50.0 µg mL^−1^. Separate 10.0 mL aliquots of this solution were transferred into different stoppered bottles and mixed with 10.0 mL of 0.1 M hydrochloric acid and 1.0 M sodium hydroxide for acidic and alkaline degradation studies.

To study acidic degradation, flasks were placed in a thermostatic oven at the following temperatures: 90.0, 80.0, 70.0, 60.0, 50.0, 40.0, and 30.0 °C. Similarly, for alkaline degradation studies, the flasks were placed in the oven at 60.0, 55.0, 50.0, 45.0, 40.0, 35.0, and 30.0 °C. After specific intervals (1, 2, 4, 6, 8, 10, and 12 h), 2.0 mL of each solution was transferred to a 10.0 mL volumetric flask and then neutralized to a pH of 7.0 using predetermined volumes of 0.1 M sodium hydroxide or 1.0 M hydrochloric acid. Finally, these solutions were completed to 10.0 mL using the mobile phase, filtered, and injected into the HPLC system under the optimum chromatographic conditions described. The remaining FAV concentration was calculated at each specified reagent, temperature, and time interval.

### pH Rate profile

The amount of 2.5 mg of FAV was accurately weighed and transferred separately into 50.0 mL volumetric flasks. Each flask was then diluted to volume with Britton/Robinson buffer solutions of pH values 2.0, 3.0, 4.0, 5.0, 6.0, 7.0, 8.0, and 9.0. Aliquots of 10 mL of these buffer solutions containing FAV were then transferred into stoppered bottles and placed in a thermostatic oven at 80.0 °C for varying time intervals. After the specified duration, 1.0 mL from the bottles was transferred into 10.0 mL volumetric flasks, neutralized to pH 7.0 using 1.0 M sodium hydroxide or hydrochloric acid solutions, and diluted to volume with the mobile phase. The remaining FAV concentration was calculated at each pH value and time interval.

### Preparation of tablet dosage forms

Ten tablets of Anviziram^®^ were weighed separately and then mixed and crushed into a fine powder. A portion equivalent to the average weight of one tablet (representing a dose of 200.0 mg of FAV) was accurately weighed and dispersed in 200.0 mL of the mobile phase. After careful shaking and sonication for 5 min, the sample was filtered and then diluted to a concentration of 50.0 μg mL^−1^ using the mobile phase. The same procedure was used to prepare a sample from an expired tablet batch. The R% was calculated using the average peak area of seven replicates.

## Results and discussion

### DOE and optimization of chromatographic analysis conditions

#### Screening and chromatographic variables effects

The DOE approach involves defining CMAs and CMPs, conducting runs, building models, evaluating results, and selecting and verifying the suggested optimized parameters. The primary objective is to separate FAV from impurities and degradation products simultaneously, while obtaining symmetric peaks with a high number of theoretical plates (N), efficient capacity factors, and a suitable analysis time. Trial runs were conducted to achieve this, and the CMA, CMP, and QTMP were set.

During early trial runs, it was observed that FAV and its impurities I and II exhibited adequate absorption at 323.0 nm, as shown in the UV spectrum in the supplementary material (Figure S1). Furthermore, it was found that using an aqueous mobile phase percentage of 92.0% instead of 90.0% yielded higher results for R1 and R2 on the C18 column. The CMPs were identified as the mobile phase's flow rate, water percentage, and pH.

After performing the 18 suggested runs, each response was analyzed and transformed as necessary to determine the significant factors, their interactions, and quadratic effects. The suggested chromatographic runs, including their CMPs and the obtained CMAs, are summarized in Table 2. The models generated for each CMA, along with the coefficients of the factors, were calculated (Supplementary material Table S2), indicating the sensitivity of the response to those factors. All models were significant, with p-values less than 0.05, F-values more than 0.1, non-significant lack of fit (p-values > 0.05**),** a high signal-to-noise ratio (> 4), and good regression of R^2^ and adjusted R^2^ (Both > 0.85, with their values close to each other) [42].

**Table 2:** Experimental factor levels and observed responses

Run	Critical method parameters (CMP)			Critical method attributes (CMA)					
	Flow rate	pH	Buffer %	Capacity Factor	Resolution (1)	Resolution (2)	Peak symmetry	Analysis time	Theoretical plates
1	1.2	3	92	1.35	6.90	11.70	0.85	10.1	18350
2	1.2	3	95	2.03	8.46	8.74	0.82	12.5	16388
3	1.2	3	98	3.33	10.89	2.79	0.82	15.5	17140
4	1.2	6	92	0.28	1.88	11.56	0.93	5.5	20584
5	1.2	6	95	0.39	3.84	13.79	1.36	6.7	8022
6	1.2	6	98	0.66	6.47	13.80	1.63	8.4	7928
7	1.2	5	92	0.77	0.95	3.01	2.47	5.5	3662
8	1.2	5	95	1.16	2.12	2.39	2.63	6.7	4128
9	1.2	5	98	2.04	2.90	1.82	2.88	9.0	5252
10	1.0	3	92	1.82	7.12	12.29	0.82	12.0	19517
11	1.0	3	95	2.57	9.16	10.06	0.84	14.7	20620
12	1.0	3	98	4.51	12.03	2.21	0.81	19.2	18796
13	1.0	6	92	0.55	1.54	11.84	0.85	6.3	29161
14	1.0	6	95	0.65	3.90	14.38	1.29	7.7	10152
15	1.0	6	98	1.08	7.14	14.41	1.65	10.2	10058
16	1.0	5	92	1.12	0.89	3.16	2.85	6.4	3700
17	1.0	5	95	1.58	2.09	2.62	2.92	7.8	4107
18	1.0	5	98	2.87	2.62	2.84	3.24	11.4	5600

Furthermore, the two- and three-dimensional contour plots for each CMA are shown in the supplementary Materials (Figures S3—S5). These contour plots represent the response plotted against each of the studied factors. Blue regions indicate lower values, while red areas indicate higher ones. From these data, it was found that the flow rate (factor A) decreased all CMA values, which helped reduce run time and decrease peak tailing. Its quadratic relation (A^2^) had the same impact except for R_1_. The pH (factor B) decreased K′, R_1_, analysis time, and number of theoretical plates (N) but increased R_2_ and symmetry values. On the contrary, its quadratic relation (B^2^) increased R_1_, analysis time, and N. Moreover, water% (factor C) increased all responses except R_2_ and N. Its quadratic relation (C^2^) had the opposite effect, except for R_2_ and analysis time.

The interaction between factors (AB, AC, BC) revealed that AB decreased K′ and symmetry but didn’t significantly impact R_2_ and N. On the one hand, the AC interaction increased K′ and R_2_ but didn’t affect symmetry or N. On the other hand, BC influenced all the responses, increasing R1, R2, and symmetry, thereby giving it a higher weight among the interactions.

#### Optimization and confirmation

**Fig. 2:**
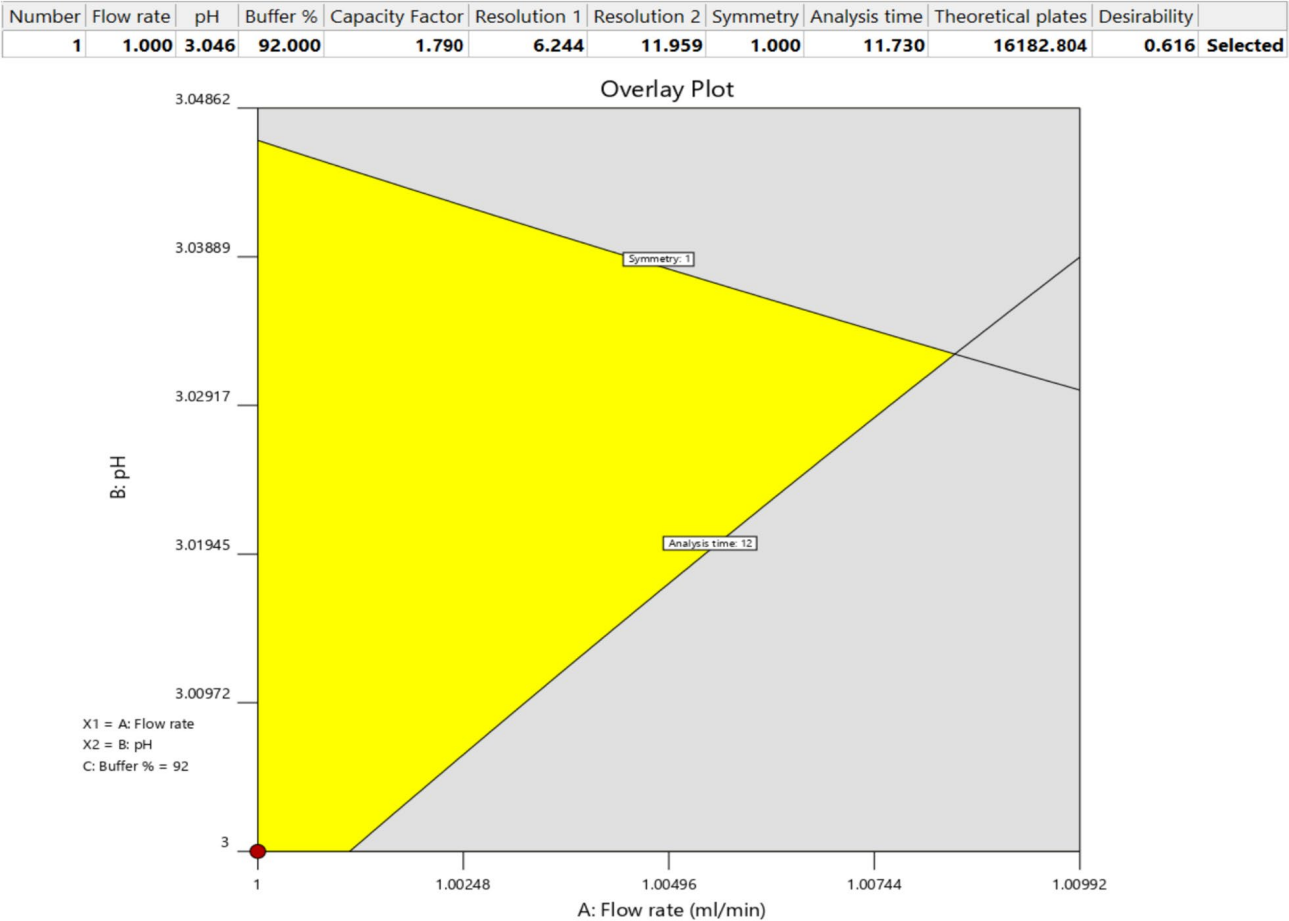
The Numerical table and overlay plot solutions to confirm the DOE's suggested chromatographic conditions

Numerical and graphical techniques were utilized to achieve the QTMPs specified earlier. For numerical optimization, the minimum values were adjusted as follows: K′ to 1.5, R_1_ and R_2_ to 4.0, and N to 4000.0. Next, the targets were set to achieve the maximum K′, R_1_, R_2_, and N with symmetry equal to 1.0 in minimal run time (Fig. 2). The suggested solution that met these criteria was ACN: Water (8.0: 92.0, v/v) mobile phase adjusted to pH 3.04 with a 1.0 mL min^−1^ flow rate. The desirability was 0.616, and the predicted run time was 11.73 min. For graphical optimization, overlay plots (Fig. 2) were used to select the working points, where areas meeting the optimization criteria are colored yellow and align with the numerical solution.

**Fig. 3:**
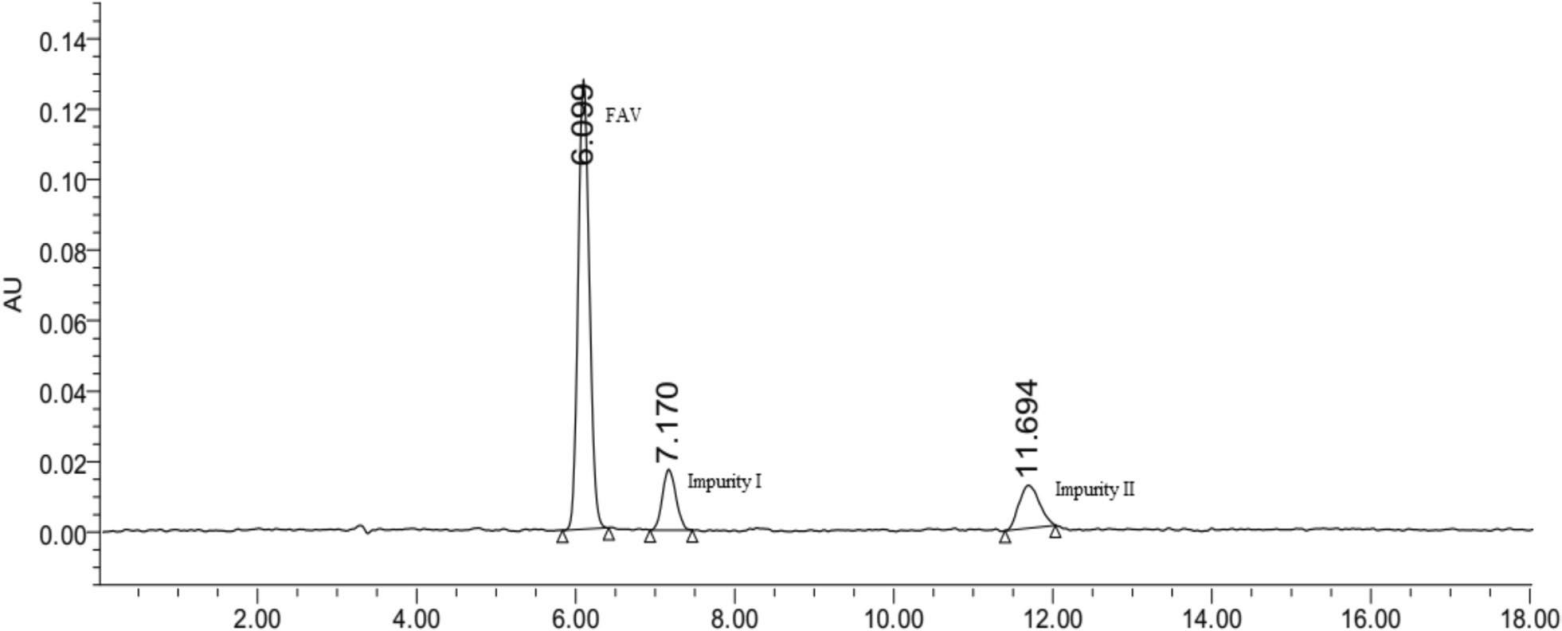
A Chromatogram showing FAV separation from its process related impurities I&II under the DOE's suggested chromatographic conditions. Optimum conditions: Mobile phase; acetonitrile and 25 mM phosphate buffer pH 3.04 at 8:92, v/v; Flow rate 1.0 mL min-1, and column RP-C18 temperature 25 °C

Runs were performed using the suggested CMPs to confirm the solution as shown in the upper table in Fig. 2. The optimal suggested conditions were a mobile phase of 25 mM phosphate buffer, pH 3.045, and acetonitrile at a ratio of 92:8 (v/v), set at a flow rate of 1.0 mL min^−1^. The runs corresponded with the predicted values, with a runtime of less than 12 min, symmetrical FAV peak, and well-resolved impurity peaks. These chromatographic conditions were ultimately used. Figure 3 displays the separation of FAV together with its impurities I and II under the suggested optimum chromatographic conditions (shown in Fig. 2), demonstrating the models' effectiveness in predicting and optimizing the method.

### Validation of the analytical methodology

After selecting the optimal separation conditions that are capable of resolving the specified impurities, the method was validated for the analysis of FAV using the ICH guidelines [13].

**Fig. 4:**
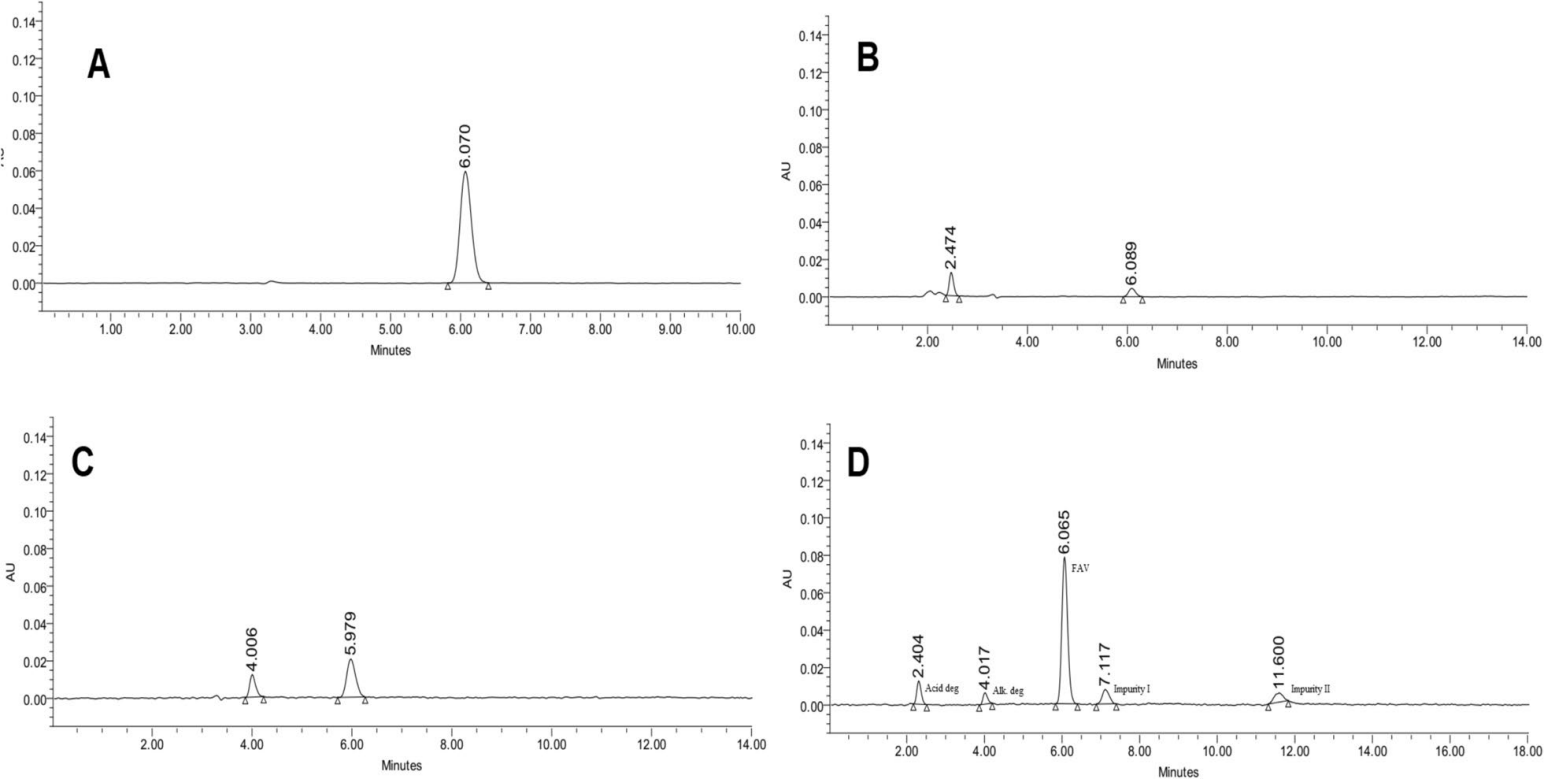
Chromatograms for FAV (**A**), its acidic degradation (**B**), alkaline degradation (**C**), and combined degradation products, FAV, together with impurities I and II (**D**)

The specificity and selectivity of the proposed method were determined. The method’s capability to resolve FAV from its manufacturing-specified impurities was proven as shown in Fig. 3. Then, the acidic and alkaline degradants were injected under the proposed chromatographic conditions. Figure 4 illustrates the separation between FAV, specified impurities, acidic and alkaline degradants, demonstrating the stability-indicating and specificity efficiency of the proposed method. Under optimized conditions, retention times were 2.40 min for the acid degradate, 4.02 min for the alkaline degradate, 6.07 min for favipiravir, 7.12 min for impurity I, and 11.60 min for impurity II, with a standard deviation of ± 0.05 for these times. It is also worth recalling that the two process impurities (I and II) are not produced through acid/base hydrolysis of Favipiravir; instead, they originate from the manufacturing process.

Table 3 summarizes the validation parameters, including linearity and range, accuracy, precision, LOD, and LOQ. Six serial concentrations of FAV within a concentration range of 5.0 to 100.0 µg mL^−1^ were injected and their average responses were recorded. The data were used to establish the linear regression and calculate the regression equation and coefficient, which are presented in Table 3. After proving the linear regression, the LOD and LOQ were calculated using the obtained data. LOD and LOQ were based on the response standard deviation (σ) and the slope (S) using the Eqs. 3.3 σ/S and 10 σ/S, respectively. The obtained values (Table 3) confirm the sensitivity of the proposed method. The system suitability parameters were 1.81, 6.6, 12.1, 16249.9, and 0.99 for K′, R1, R2, N, and symmetry, respectively.

**Table 3:** Analytical method validation and system suitability data for determination of FAV under the proposed chromatographic conditions

Range (µg mL^−1^)	Equation	R^2^	Accuracy* (recovery% ± RSD%)			Precision* (recovery% ± RSD%)						LOD (µg mL^−1^)	LOQ (µg mL^−1^)
						Repeatability (µg mL^−1^)			Intermediate precision (µg mL^−1^)				
			25.00	50.00	70.00	10.00	50.00	90.00	10.00	50.00	90.00		
5.00–100.00	y = 73504x–55168	0.9999	101.4 ± 0.01	101.6 ± 0.04	99.7 ± 0.81	99.7 ± 0.13	101.2 ± 0.07	102.3 ± 0.003	101.7 ± 1.4	100.6 ± 0.04	102.4 ± 0.33	0.51	1.54

System suitability parameters				
FAV capacity factor ( K′)	Resolution 1 (FAV/impurity I)	Resolution 2 (impurity I/II)Resolution 2 (impurity I/II)	FAV number of theoretical plates (N)	Symmetry factor
1.81	6.6	12.1	16249	0.99

The accuracy was then assessed. Three QC standards spiked within the placebo solution containing the excipients were injected three times, and the recovery percentages were calculated to confirm the close agreement with the true values (Table 3). To establish precision, three other QC samples covering the low, middle, and high ranges were injected on the same day to assess repeatability and on three different days to evaluate intermediate precision. The R% ranged from 99.7 to 102.3%, with a RSD% of less than 2%. This illustrates the closeness of the calculated concentrations to the actual concentrations and the consistency of the results. For specificity, the blank and placebo chromatograms exhibited no peaks at the retention times corresponding to FAV, impurities, or degradates. Furthermore, the purity angle for the FAV peak in all stressed samples was found to be less than the purity threshold, indicating the homogeneity of the peak and thereby confirming the absence of co-eluting impurities. Additionally, when degradates, Impurity I and II, were spiked into a tablet excipient mixture, they were recovered without any shift in retention time or appearance of new peaks. Changing the pH to 2.9 and 3.1 (3.04 ± 0.1) demonstrated a R% for favipiravir ranging from 99.3% to 100.8%, with a relative standard deviation results (RSD%) of 0.76. Additionally, all QTMP were retained, indicating enhanced robustness within the method’s design space.

Solution stability studies showed that favipiravir’s peak area remained within the range of 98.5% to 101% of the initial value after 24 h, with no emergence of new peaks. The recoveries of Impurities I and II ranged from 99 to 102% relative to the initial measurements. Therefore, it can be concluded that the solutions are stable for a minimum of 24 h under ambient conditions. Similar results were observed when refrigerated stock solutions were analyzed and compared to freshly prepared standards.

### Kinetic investigation and pH rate profile

The proposed study focused on the hydrolytic degradation pathways of FAV, being a carboxamide derivative, where it’s particularly prone to hydrolysis. Hence, the focus was directed towards the pH degradation rate studies to find the most stable pH, which has not been reported yet. Moreover, it has already been reported by Marzouk et al*.* that FAV remains stable under the photolytic and thermal stress conditions [24]. The proposed study investigated the degradation kinetics of FAV in 1 M sodium hydroxide and 0.1 M hydrochloric acid. The reason for choosing a different strength for sodium hydroxide (higher) was that the decomposition rate of FAV was too slow to obtain reliable kinetic data at lower strengths of sodium hydroxide. The degradation process was found to follow pseudo-first-order kinetics at the selected temperatures (30.0, 40.0, 50.0, 60.0, 70.0, 80.0, and 90.0 °C) for acidic degradation, and at temperatures (30.0, 35.0, 40.0, 45.0, 50.0, 55.0, and 60.0 °C) for alkaline degradation. The Britton/Robinson buffer was used to cover the entire pH range and prevent any potential effects of using different buffer species. Moreover, the pH values were checked both before and after the reaction and were found to be stable and unchanged. The concentration of intact FAV, determined by the proposed HPLC method, regularly decreased over time, as shown in the supplementary material Figure S6. The straight-line slopes were used to calculate the apparent first-order degradation rate constant and the half-life at each temperature for the acidic and alkaline degradation processes, shown in Table 4. The apparent first-order degradation rate constant and the half-life for each pH value at 80.0 °C are also shown in Table 4. In acidic and alkaline conditions, the degradation rate increased markedly with rising temperature, as evidenced by steeper slopes in the kinetic plots and higher calculated rate constants (Kobs). Additionally, at higher temperatures, favipiravir exhibited significantly shorter half-lives (t_1/2_), indicating reduced stability. Similarly, the stability of favipiravir varied notably across the tested pH range, highlighting the necessity of meticulously regulating pH and temperature to maintain drug integrity. Furthermore, the safety data sheets for favipiravir specify that it is a flammable solid and may cause skin and serious eye irritation, in addition to potential respiratory irritation; therefore, safe handling is required. Hence, these findings demonstrate the substantial influence of environmental conditions on the stability of favipiravir, providing essential guidance for its formulation, storage, and analytical quantification. The degradation mechanism probably resembles that reported by Hoda M. Marzouk et al. [24], where the pathway begins with the hydrolytic cleavage of the amide group under both acidic and alkaline conditions. This process yields a free amine and the corresponding carboxylic acid, 6-fluoro-3-hydroxy-2-pyrazinecarboxylic acid. Under acidic conditions, this acid undergoes further transformation: the protonation of the carbonyl group, which enhances its electrophilicity and promotes dehydration and the formation of a resonance-stabilized acylium cation, specifically the 6-fluoro-3-hydroxypyrazin-2-yl acylium ion. Future research initiatives may involve molecular docking comparisons of favipiravir, its active form, related impurities, and degradation products, including 6-fluoro-3-hydroxy-2-pyrazinecarboxylic acid and the 6-fluoro-3-hydroxypyrazin-2-yl acylium ion. Such comparative docking analyses could provide significant insights into the binding affinities of favipiravir and its transformation derivatives, thereby advancing comprehensive risk assessment and potentially identifying novel pharmacophores [43].

**Table 4:** Degradation rate constant (Kobs) and half-life (t_1/2_) of FAV in 0.1 M hydrochloric acid, 1 M sodium hydroxide, and Britton/Robinson buffer solutions

	Temperature (°C)	K_obs_	t_½_
Hydrochloric acid	30.0	0.0168119	41.2
	40.0	0.0260239	26.6
	50.0	0.0499751	13.9
	60.0	0.1034047	6.7
	70.0	0.1418648	4.9
	80.0	0.329329	2.1
	90.0	0.5149508	1.3
Sodium hydroxide	30.0	0.005527	125.38
	35.0	0.005297	130.83
	40.0	0.01543	44.91
	45.0	0.017042	40.66
	50.0	0.024872	27.86
	55.0	0.034315	20.20
	60.0	0.041915	16.53
Britton/Robinson buffer	pH		
	2.0	0.2004	3.459
	3.0	0.0599	11.574
	4.0	0.0106	65.416
	5.0	0.0001	6018.237
	6.0	0.0005	1504.559
	7.0	0.0012	601.824
	8.0	0.0014	501.520
	9.0	0.0016	429.874

**Fig. 5:**
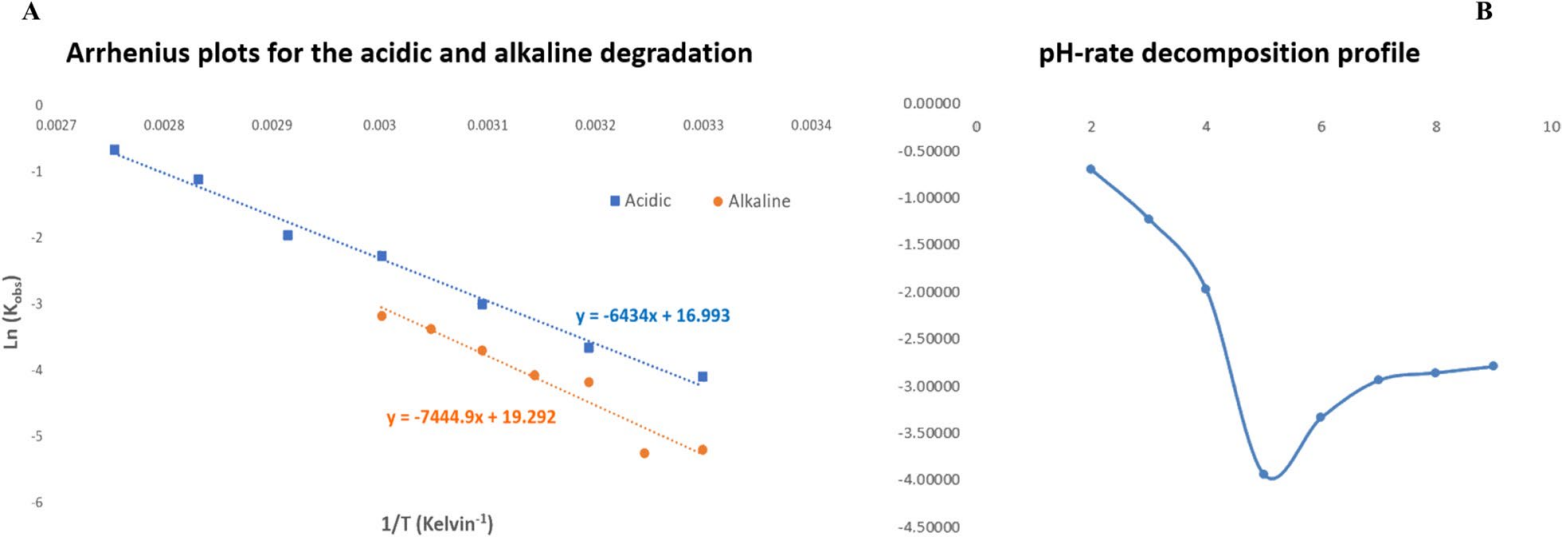
Arrhenius plots for the degradation of FAV in 0.1 M hydrochloric acid (Blue ■) and 1 M sodium hydroxide (Orange ●) as determined using the developed method (**A**) together with the pH-rate profile for FAV decomposition in Britton/Robinson buffer at 80 °C (**B**)

Figure 5 shows the Arrhenius plots and pH-rate degradation profile of FAV in Britton/Robinson buffer solutions. Arrhenius plots were linear, and the activation energies for the acidic and alkaline degradation processes were calculated to be 53492.276 kJ/mol and 61896.899 kJ/mol, respectively. The pH-rate profile of FAV degradation in Britton-Robinson buffer solutions indicates that it is most stable at a pH of 5.0. Notably, Marzouk et al. previously reported on the stability of favipiravir under acidic (0.1 N HCl) and alkaline (0.5 N NaOH) conditions at room temperature for 12 h [24]. They determined the pseudo-first-order rate constants (Kobs) to be 0.225 h-1 for acid hydrolysis and 0.158 h-1 for alkaline hydrolysis, with corresponding half-lives (t1/2) of approximately 3.08 h and 4.39 h, respectively. In our study, the degradation kinetics of favipiravir were investigated over a broader temperature range (30–90 °C) for acidic conditions and (30–60 °C) for alkaline conditions, revealing temperature-dependent stability profiles based on the calculated activation energies. These findings complement and extend the existing literature, providing deeper insight into how temperature affects the hydrolytic stability of favipiravir within a pH range, and offer better predictive modeling of its shelf life under varying storage conditions.

### Method’s application

The proposed method was applied to estimate FAV in commercial tablets (Anviziram^®^). The results of (R% ± RSD%) of 7 replicates were found to be (102.91 ± 1.43), agreeing with the labelled claims. Another Anviziram^®^ expired tablet batch was analyzed to check the stability of the drug after its expiration date and confirm the stability-indicating capability of the proposed method. The recovery% were found to be 100.79 ± 1.09 (*n* = *7*). The chromatogram of the expired Anviziram^®^ tablets’ batch showed a degradation product peak (Supplementary Material Figure S7), which is compatible with the alkaline degradation product peak (Fig. 4D).

###  Greenness and blueness assessment and comparison to other reported methods

The development of eco-friendly analytical methods has increased over the years. Greenness assessment in analytical methodologies is increasingly recognized as a critical factor for sustainable development in scientific research. Its primary concerns were operator safety, environmental impact, and sustainability [44]. From this perspective, the greenness of the developed method was calculated and compared with seven reported chromatographic methods using the AGREE metric. AGREE metric is a clock-shaped chart where segments on the side represent each of the Green analytical principles, and the method’s overall score is located in the center [40, 45]. The proposed method assigned penalty points were for the offline analysis and handling (Segments 1 and 3), waste volume of 12 mL (calculated as runtime multiplied by flow rate in Segment 7), analysis throughput (with a total of 5 analytes and a sample throughput of 60/12 = 5 samples per hour in Segment 8), the energy of utilizing HPLC (Segment 9), the consideration that not all reagents are biobased due to the use of ACN (Segment 10), the volume of ACN employed in the method (8% multiplied by 12 min equating to 1 mL in Segment 11), and ACN being highly flammable (Segment 12). Table 5 shows that one method [23] obtained a significantly higher score as it did not involve organic solvents, unlike the 8% acetonitrile employed in this study. However, that method only measured FAV with its acidic degradation product, whereas the proposed method assessed FAV, acidic and alkaline degradation products, and two impurities. Additionally, the AGREE score calculated for the reported method was 0.76, which is slightly lower than the reported 0.84. This difference is mainly attributed to AGREE’s third segment. The reported score considered the method online while the samples were transported, prepared, and then analyzed, making it an offline measurement. Furthermore, some degradation studies have reported the use of greener solvents. However, they still achieved similar [24] or lower scores [27, 32] due to the proposed method's high throughput and high water percentage in the mobile phase (92%), resulting in a lower overall ecological impact. The reported impurity studies [19, 29] measured FAV with more than two impurities, and Impurity (II) was found to be mutual among the three methods. This impurity was detected in 5.4 min by the LC–MS method, which consumes more energy than the LC-UV method, and in 11.6 min by the reported PDA method. Both methods used gradient elution containing ACN and had an average run time of 45 min. In contrast, the proposed method used isocratic elution and detected impurity (I), which is involved in more synthesis pathways than impurity (II).

**Table 5:** Comparison between the proposed and some reported chromatographic methods using the AGREE & BAGI metrics

	Proposed method	Reported method [28]	Reported method [24]	Reported method [32]	Reported method [31]	Reported method [27]	Reported method [19]	Reported method [29]
Detector	PDA	PDA	PDA	UV	UV	UV	PDA	MS
FAV Linearity (μg ml − 1)	5.00–100.00	5.00–100.00	6.25–250.00	0.50–100.00	49.80–150.00	2.00–12.00 & 20.00–60.00	0.100–1499.99	0.50–1.00
Organic solvent	8%ACN*	Free	28%MeOH & 10%ACN*	50% ACN*	25%MeOH*	25%MeOH & 35%EtOH*	100% ACN* in gradient	100% ACN* in gradient
Run time (minutes)	12.00	8.00	5.50	7.00	7.00	10.00	40	50.00
Column	C18	C18	C18	C18	C18	C18	C18	C18
LOD (μg ml − 1)	0.51	1.379	1.02	0.037	0.15	0.18	0.04	-
LOQ (μgml − 1)	1.54	4.179	3.10	0.122	0.31	0.55	0.10	0.50
Aim	Impurities and Stability assessment in dosage form	Acidic stability assessment in dosage form	Stability assessment in dosage form	Stability assessment and In‑vitro safety evaluation	Stability assessment in dosage form	Stability assessment in bulk and dosage form	Impurities assessment in bulk	Impurities assessment and Pharmacokinetic study in rat plasma
AGREE								
MoGAPI								
BAGI								

^*^ACN stands for Acetonitrile, MeOH for Methanol, and EtOH for Ethanol

The BAGI approach [41] is a newly developed, user-friendly, free software that considers ten features to assess the practicality and feasibility of analytical techniques. The output is presented as an asteroid pictogram divided into ten segments, with a score in the center. Each segment is colored dark blue, blue, light blue, or white to indicate high, medium, low, or no adherence to the method’s applicability. BAGI recommends that a method with a final score of at least 60 be considered practical. The proposed HPLC method achieved a score of 85.0, demonstrating its practicality. The lowest score was 77.5, reported by the method [24], which utilized LC–MS (BAGI segment 3) and a long run time (BAGI segment 6). Still, it is considered practical, as the score was over 60.

Similar to AGREE, the Green Analytical Procedure Index (GAPI) uses colored pentagrams to visually evaluate the environmental friendliness of analytical methods. However, GAPI doesn't provide a total numerical score, making it hard to compare methods objectively based solely on colors. To overcome this, a modified version called Modified GAPI (MoGAPI) was recently developed, incorporating numerical scores aligned with the Eco-Scale metric. The scoring differences between MoGAPI and AGREE mainly stem from their assessment criteria. MoGAPI considers factors like extraction scale (Segment 6), solvent type (Segment 7), solvent hazards (Segments 10 and 11), energy use per run (Segment 12), and waste treatment (Segment 15). Conversely, AGREE emphasizes the number of analytes per run, method throughput (Segment 8), and automation level (Segment 5). These perspectives are illustrated in Table X, where methods with identical MoGAPI scores show differences in the AGREE scores. These metrics don’t conflict, but rather complement each other, offering a more comprehensive view of the greenness of each method. MoGAPI's strengths are especially clear in Segment 7, where solvent-free methods (e.g., Yasmin et al.) are marked green, ethanol-based methods (Chakraborty et al.) are yellow, and methods using conventional organic solvents are penalized with red. Additionally, MoGAPI's detailed evaluation of solvent hazards, based on NFPA classifications, further distinguishes methods. For example, Yasmin et al.'s micellar systems showed a clear advantage in Segment 11 due to lower flammability compared to traditional solvents. Interestingly, methanol, though penalized in Segment 7 for not being green (Patel et al.), was recognized for lower health hazards (Segment 10) than solvents like acetonitrile and ethanol. The differences in runtime between methods in MoGAPI’s assessment can be interpreted through segments addressing energy consumption (Segment 12, calculated as Power [kW] × Time [h]) and waste generation (Segment 14, calculated as flow rate × runtime). For instance, Mangukiya et al.'s HPLC–PDA method scored lower in these segments due to its longer runtime compared to the other methods using HPLC–UV. Similarly, Koganti et al. had comparable runtime but incurred additional penalties in Segment 12 because of higher energy needs for the MS detector, even though waste generation (Segment 14) was similar. The proposed method scored 70 due to offline analysis and handling (Segment 1 and 3), the absence of green solvents (Segment 7), health and safety hazards (Segment 10 and 11), waste volume exceeding 10 mL (Segment 14), and the lack of waste treatment options (Segment 15).

## Conclusion

In this study, a robust, environmentally friendly, and Quality-by-Design (QbD)-optimized High-Performance Liquid Chromatography (HPLC) method was developed, capable of simultaneously separating and quantifying Favipiravir (FAV), in presence of two primary manufacturing impurities, and its hydrolytic degradation products. Through comprehensive degradation kinetic investigations, the method effectively elucidated the stability profile of FAV, identifying that optimal drug stability is achieved at a pH of 5.0. Moreover, activation energies were calculated for acidic and alkaline hydrolytic degradation pathways, offering critical insights into temperature-dependent drug stability and shelf-life estimations. The validated analytical method demonstrated excellent specificity, accuracy, precision, and robustness, rendering it suitable for routine quality control analysis of FAV in pharmaceutical formulations, with no interference from excipients. Its practical applicability was confirmed through successful analysis of marketed tablets and the identification of degradation products in expired dosage forms, thereby underscoring its significant value as a stability-indicating assay. Furthermore, the environmental implications of the analytical procedure were assessed utilizing established green analytical chemistry metrics, resulting in a commendable Analytical Greenness (AGREE) score of 0.65 and an exemplary Blue Applicability Grade Index (BAGI) score of 85.0. This demonstrates that the methodology not only meets the requisite analytical performance standards but also adheres to the principles of sustainability and practicality. Future studies could expand on these results by employing LC–MS degradation profiling, as well as performing molecular docking and computational analyses to compare the binding affinities of FAV, its active metabolites, impurities, and degradation products. These investigations might deepen our understanding of how drug degradation and impurity formation affect antiviral properties. Moreover, further use of this approach could help establish a standardized pharmacopeial monograph for FAV, assist in impurity profiling in new dosage forms, and support monitoring in emerging therapeutic areas, such as combination therapies for resistant viral infections or cancer treatments.

## Supplementary Information

Supplementary Material 1.

## Data Availability

The original contributions presented in the study are included in the article; further inquiries can be directed to the corresponding authors.

## Abbreviation

COVID-19: Coronavirus disease 2019
SARS CoV-2: Severe acute respiratory syndrome coronavirus 2
FAV: Favipiravir
FAV-RTP: Favipiravir ribofuranosyl-5′-triphosphate
ICH: International council for harmonization
Impurity I: 3,6-dichloro pyrazine-2-carbonitrile
Impurity II: 6-Fluoro-3-hydroxypyrazine-2-carbonitrile
OFAT: One factor at A time
DOE: Design of experiments
QbD: Quality by design
AGREE: Analytical greenness metric
BAGI: Blue applicability grade index
HPLC: High-performance liquid chromatography
PDA: Photodiode array detector
CMA: Critical method attributes
CMP: Critical method parameters
QTMP: Quality target method profile
Rs: Resolution
K′: Capacity factor
N: Number of theoretical plates
LOD: Limit of detection
LOQ: Limit of quantification
QC: Quality control
R%: Recovery percentage recovery
RSD%: Relative standard deviation percentage
