# Supplementary material for: A quality-by-design optimized LC method for navigating degradation kinetics and quantification of favipiravir in the presence of degradation products and manufacturing impurities
Source: BMC Chem. 2025 Aug 15;19(1):244. doi: 10.1186/s13065-025-01610-2 (PMC12357339; doi:10.1186/s13065-025-01610-2)
Supplement: Supplementary file 1 — Supplementary Material 1. [file 13065_2025_1610_MOESM1_ESM.pdf]

Supplementary Material

A Quality-by-Design Optimized LC Method for Navigating Degradation Kinetics and Quantification of Favipiravir in the presence of Degradation Products and Manufacturing Impurities

Adel Ehab Ibrahim[1,2*], Mohamed Farouk[3], Samy G. Alamir[1,4], Baher I Salman[5], Tarek S. Belal[6], Sami El Deeb[7*], and Ahmed Al-Harrasi[1*]

1 Natural and Medical Sciences Research Center, University of Nizwa, Birkat Al Mauz 616, Nizwa city, Oman; adel@unizwa.edu.om (A.I), aharrasi@unizwa.edu.om (A.A).

2 Pharmaceutical analytical chemistry department, Faculty of Pharmacy, Port-Said University, Port-Said 42511, Egypt

3 Nawah Scientific Inc., Mokatam, Cairo, Egypt; mohamed.farouk@nawah-scientific.com (M.F.)

4 Pharmaceutical Analytical Chemistry Department, Faculty of Pharmacy, Ain Shams University, Abassia 11566, Cairo, Egypt; sami.goerge@pharma.asu.edu.eg (S.A)

5 Pharmaceutical Analytical Chemistry Department, Faculty of Pharmacy, Al-Azhar University, Assiut branch, Assiut 71524, Egypt; Bahersalman@azhar.edu.eg (B.S)

6 Pharmaceutical Analytical Chemistry Department, Faculty of Pharmacy, University of Alexandria, Elmessalah 21521, Alexandria, Egypt; tbelaleg@yahoo.com (T.B)

7 Institute of Medicinal and Pharmaceutical Chemistry, Technische Universitaet Braunschweig, 38106 Braunschweig, Germany; s.eldeeb@tu-bs.de

* Correspondence: adel@unizwa.edu.om; s.eldeeb@tu-bs.de; aharrasi@unizwa.edu.om

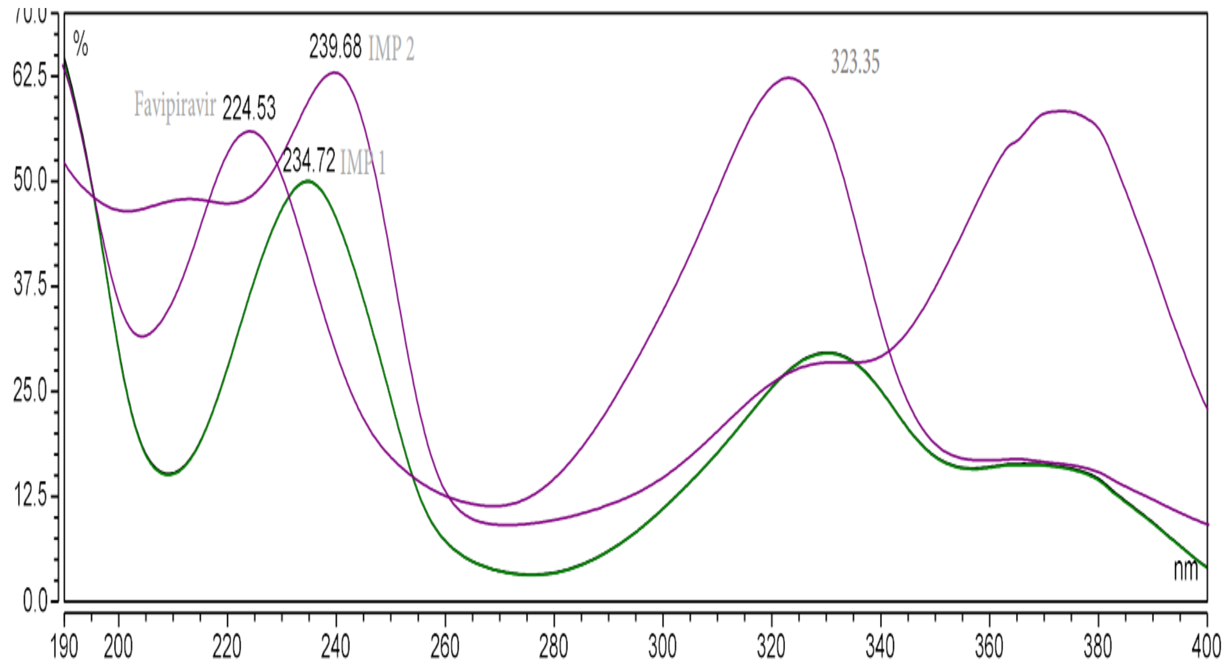

Supplementary materials Figure S1: Typical UV spectrum of Favipiravir and its pure impurities I and II.

Supplementary materials Table S2: CMAs and models generated.

CMA	Model*	Model		Lack of fit	R^2	Adjusted R^2	Adequate Precision (S/N ratio)
		P-Value	F-Value	P-Value			
**Capacity Factor**	Log (K′) = -0.082*A -0.319*B +0.191*C -0.028*AB +0.009*AC -0.012*BC -0.095*A² -0.232*B² -0.045*C² + 0.358	< 0.0001	226.207	0.363	0.996	0.992	52.595
**Resolution (1)**	Log (R_1+ 0.55) = -0.007*A -0.177*B +0.178*C +0.008*AB - 0.018*AC +0.078*BC +0.081*A² +0.396*B² -0.028*C² + 0.340	< 0.0001	340.080	0.055	0.997	0.995	55.982
**Resolution (2)**	R_2 = -0.143*A +2.666*B -1.775*C+ 0.134*AC+ 2.974*BC -1.651*A²+ 10.297*B² -1.181*C² +2.772	< 0.0001	131.990	0.502	0.992	0.984	28.461
**Symmetry**	Symmetry = -0.0375*A +0.229*B +0.152*C -0.008*AB +0.182*BC -0.230*A² -1.876*B² -0.009*C² +3.168	< 0.0001	114.980	0.491	0.990	0.982	28.359
**Analysis time**	Sqrt(Analysis time) = -0.134*A -0.502*B+ 0.366*C +0.034*AB - 0.032*AC -0.054*BC -0.248*A² +0.333*B² +0.0698*C² +3.089	< 0.0001	902.180	0.998	0.999	0.998	101.097
**Number of Theoretical plates (N)**	1/Sqrt(N) = 0.0002*A +0.001*B +0.0004*C +0.0008*BC+ 0.003*A² -0.008*B² - 0.0007*C² +0.014	0.0002	14.600	0.175	0.911	0.850	9.948

*(A) is the flow rate, (B) is pH, and (C) is the water percentage.

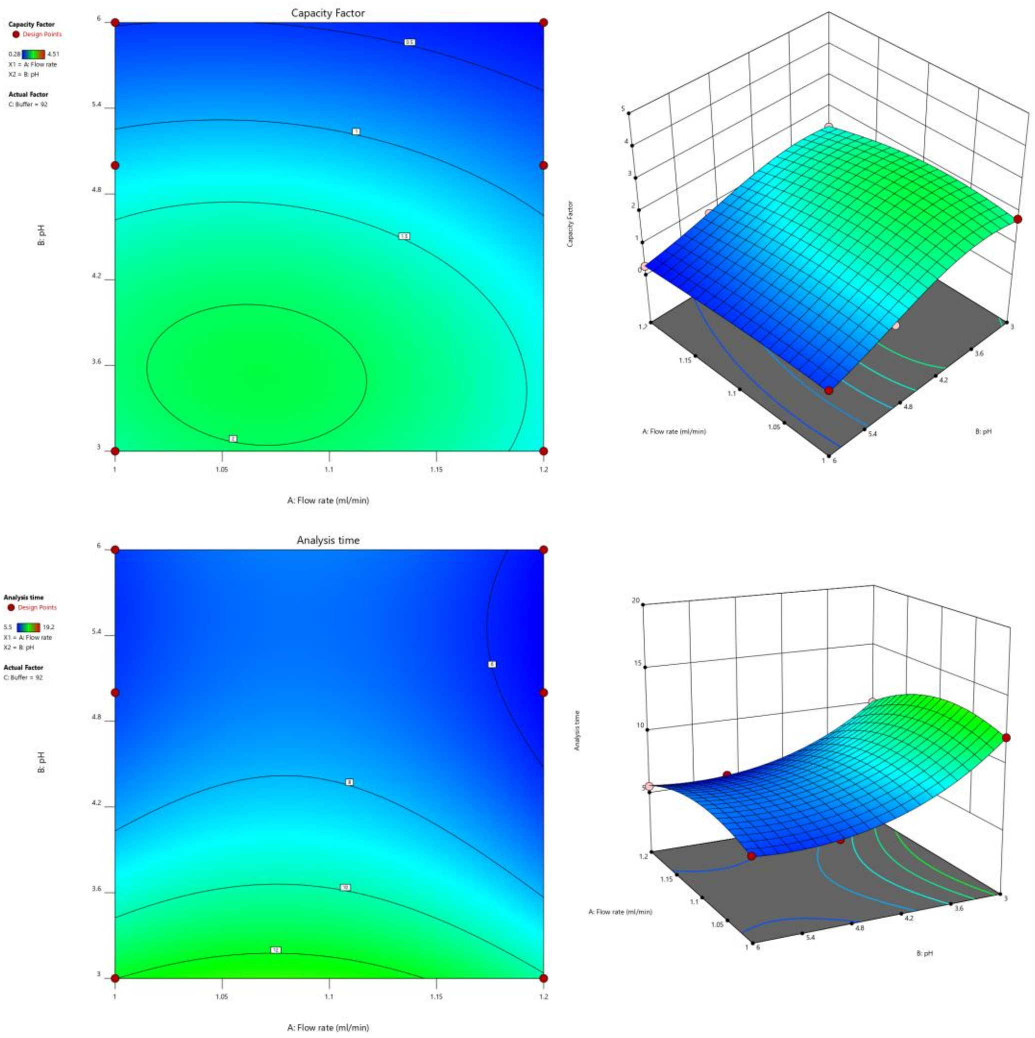

Supplementary materials Figure S3: Two and three-dimensional contour plots for the studied CQAs (Run time and K′)

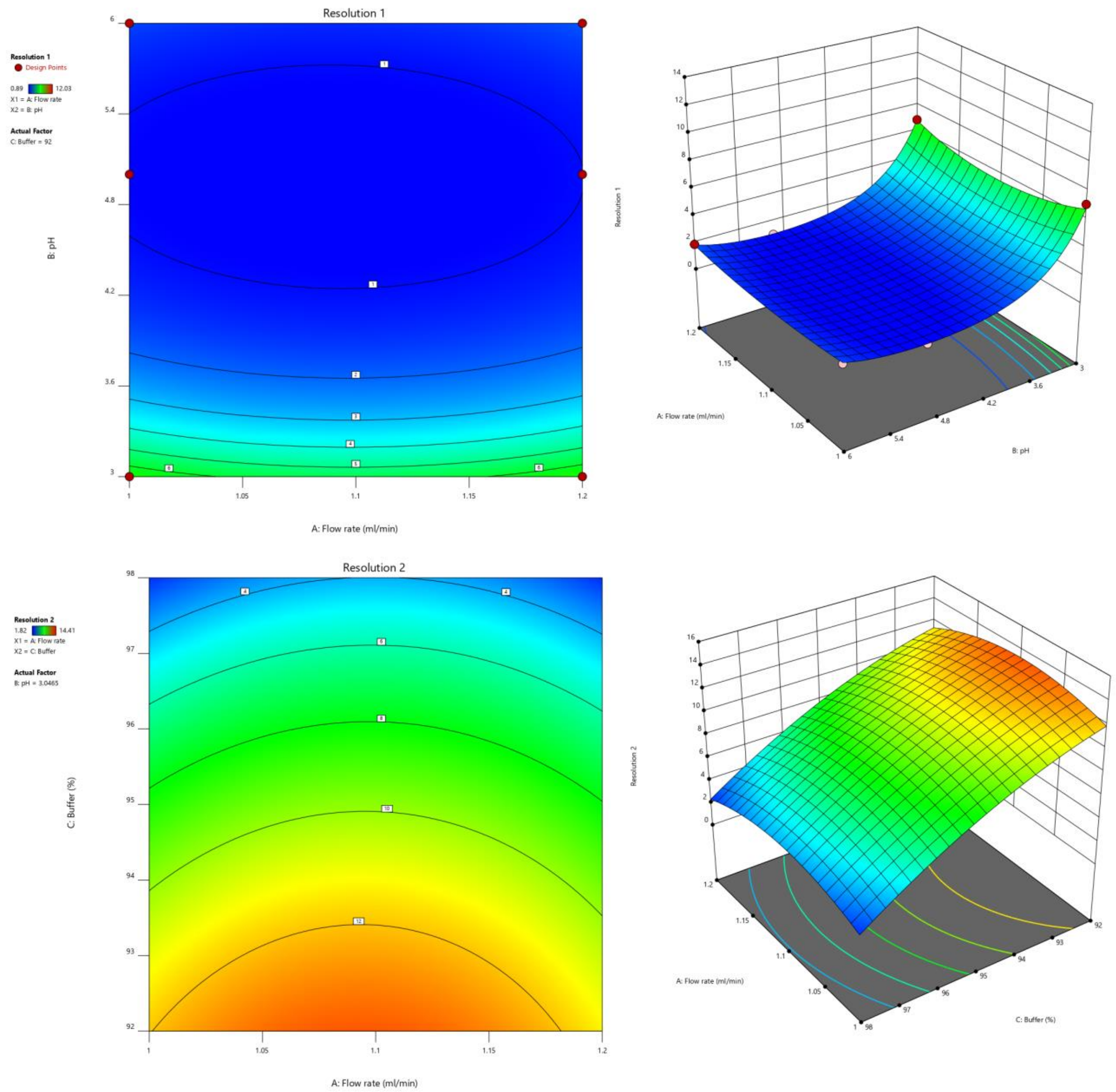

Supplementary materials Figure S4: Two and three-dimensional contour plots for the studied CQAs (resolution R_1 and R_2)

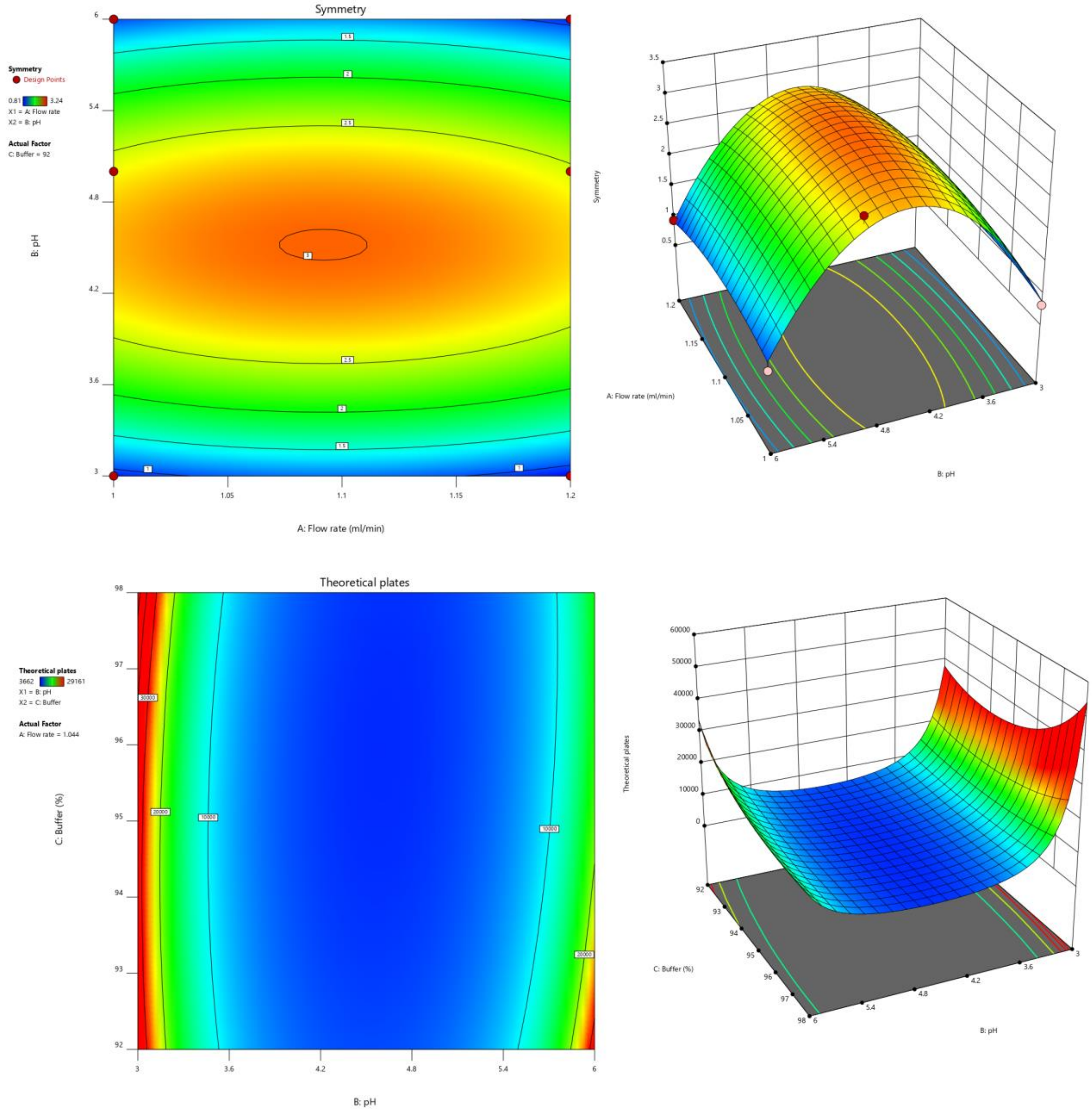

Supplementary materials Figure S5: Two and three-dimensional contour plots for the studied CQAs (FAV N and peak symmetry)

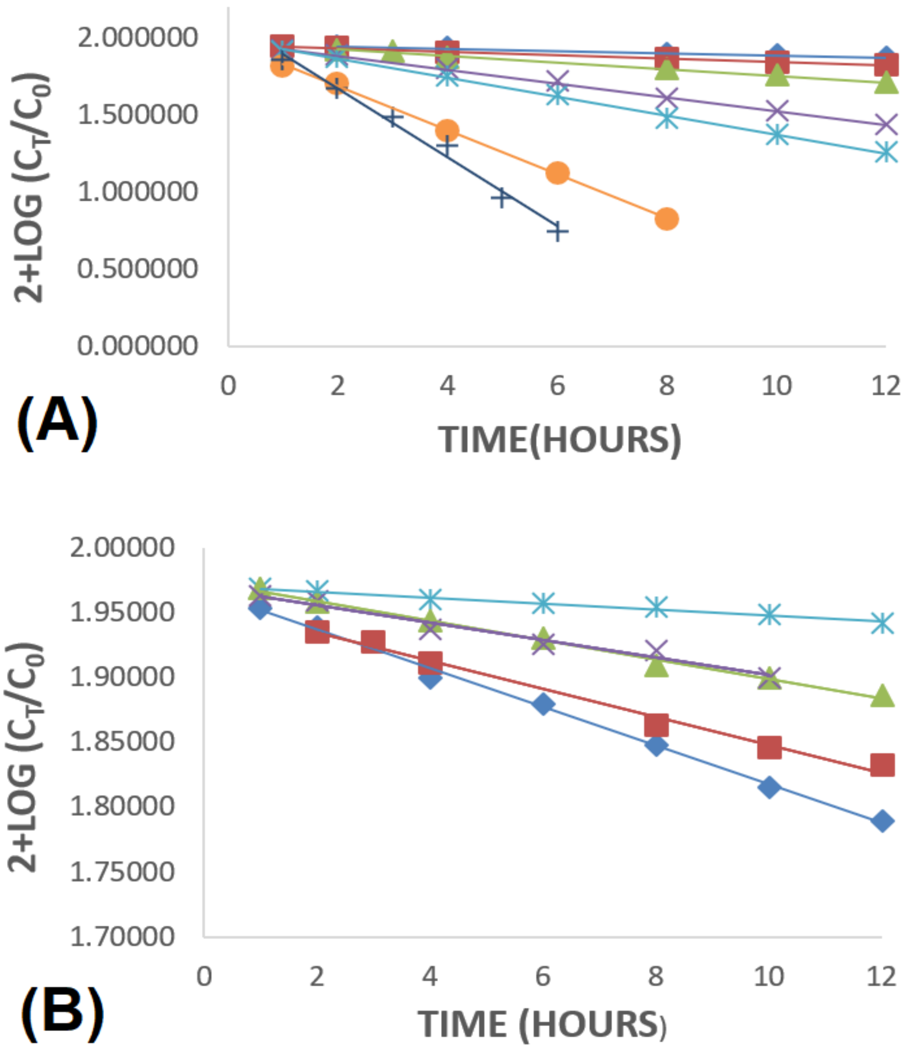

Supplementary materials Figure S6: Pseudo first-order plots for Favipiravir degradation in different conditions: (A) 0.1M hydrochloric acid and (B) 1M sodium hydroxide at various temperatures. CT represents the concentration at time t, and C0 represents the concentration at zero time.

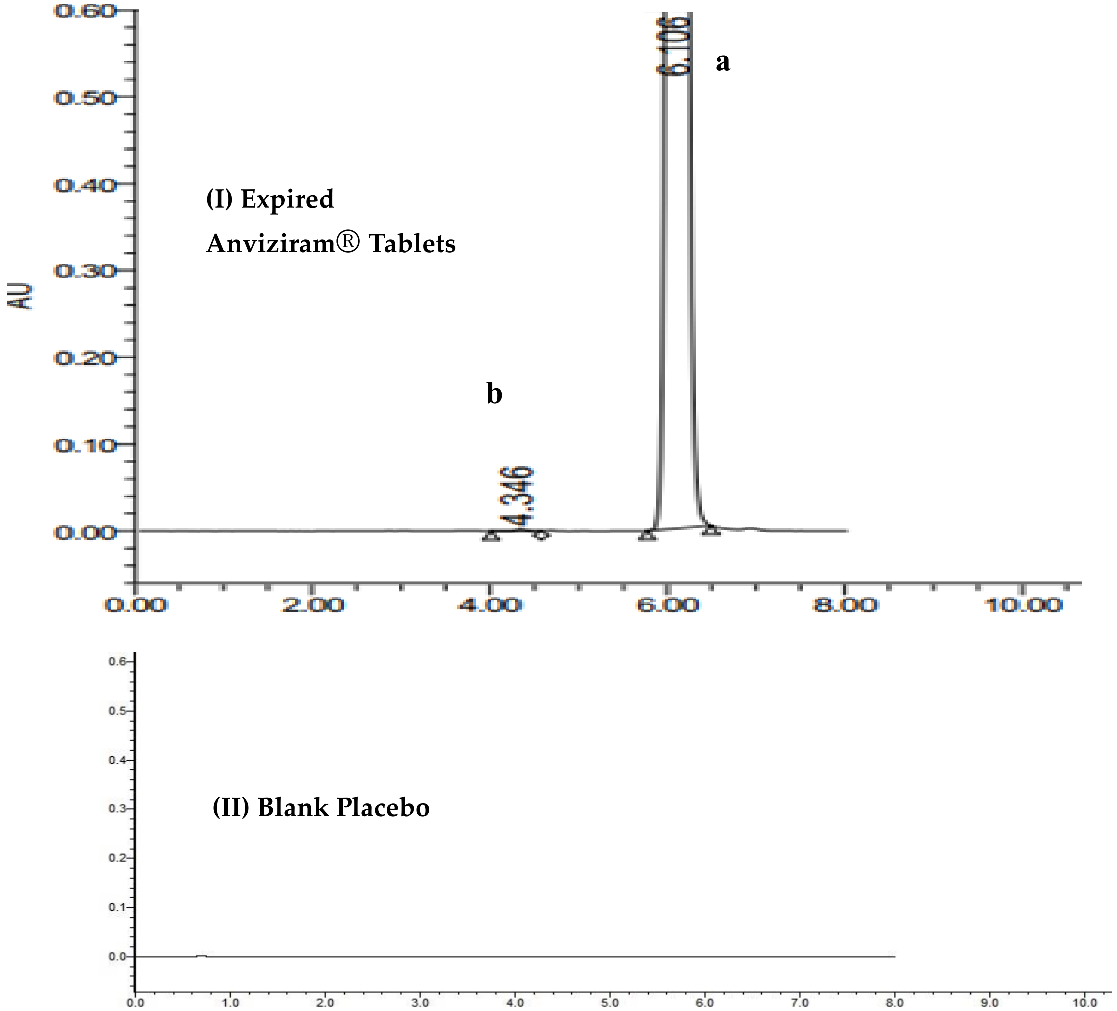

Supplementary materials Figure S7: HPLC chromatograms showing the studied expired lot of Anviziram® tablets (I) containing FAV (a), which were found to show a degradation product (b), together with a blank placebo (II) injected to show absence of any excipient's interferences.
